# Trends in the Diversity, Distribution and Life History Strategy of Arctic Hydrozoa (Cnidaria)

**DOI:** 10.1371/journal.pone.0120204

**Published:** 2015-03-20

**Authors:** Marta Ronowicz, Piotr Kukliński, Gillian M. Mapstone

**Affiliations:** 1 Marine Ecology Department, Institute of Oceanology, Polish Academy of Sciences, Sopot, Poland; 2 Life Science Department, Natural History Museum, London, United Kingdom; The Evergreen State College, UNITED STATES

## Abstract

This is the first attempt to compile a comprehensive and updated species list for Hydrozoa in the Arctic, encompassing both hydroid and medusa stages and including Siphonophorae. We address the hypothesis that the presence of a pelagic stage (holo- or meroplanktonic) was not necessary to successfully recolonize the Arctic by Hydrozoa after the Last Glacial Maximum. Presence-absence data of Hydrozoa in the Arctic were prepared on the basis of historical and present-day literature. The Arctic was divided into ecoregions. Species were grouped into distributional categories according to their worldwide occurrences. Each species was classified according to life history strategy. The similarity of species composition among regions was calculated with the Bray-Curtis index. Average and variation in taxonomic distinctness were used to measure diversity at the taxonomic level. A total of 268 species were recorded. Arctic-boreal species were the most common and dominated each studied region. Nineteen percent of species were restricted to the Arctic. There was a predominance of benthic species over holo- and meroplanktonic species. Arctic, Arctic-Boreal and Boreal species were mostly benthic, while widely distributed species more frequently possessed a pelagic stage. Our results support hypothesis that the presence of a pelagic stage (holo- or meroplanktonic) was not necessary to successfully recolonize the Arctic. The predominance of benthic Hydrozoa suggests that the Arctic could have been colonised after the Last Glacial Maximum by hydroids rafting on floating substrata or recolonising from glacial refugia.

## Introduction

The class Hydrozoa is a monophyletic group in the phylum Cnidaria, consisting of approximately 3500 currently described species [1]. The basic life cycle of hydrozoans comprises a succession of developmental stages: planula larva, hydroid and dioecious hydromedusa [2]. Sessile polyps produce medusae through asexual budding. A medusa is a mobile reproductive stage. After fertilisation a planula larva develops. In about 70% of species a free swimming medusa is suppressed [3,4]. In this case, the medusa does not leave the hydroid colony but gonophores are maintained on the polyp as sporosacs (fixed gonophores). The loss of the medusa is recognised as an evolutionary process [4,5]. The medusa stage can be reduced to medusoids, intermediary forms liberated from the polyp but short-lived, that disperse gametes over a short distance. There is also a group of species that completely lack a benthic stage (e.g. Trachymedusae, Narcomedusae, Siphonophorae). Planula larvae of benthic species live from hours to up to 20 days as given by Cornelius and Sommer and references cited therein [6,7]. They swim or crawl to a suitable substrate where they settle and metamorphose into a new polyp stage. Little is known about longevity and dispersal potential of planulae. We assume, after Jackson & Coates [8], Hughes [9] and Pagliara et al. [10], that lecithotrophic, non-feeding planula larvae are rather short-lived and possess restricted dispersal potential.

The Hydrozoa are a potentially good model for testing hypotheses about dispersal and the colonization of new regions because they have different dispersal strategies related to different life histories. Many Hydrozoa with circumglobal distributions are benthic [11] which may indicate that having medusa stage is not necessary for hydrozoan taxa to colonize distant places.

The Arctic region is a geologically young system and provides an opportunity for examination of dispersal. In recent geological history (back to Quaternary glaciations), the global sea level has fluctuated approximately 100 m between glacial lowstands and inter-glacial highstands [12,13]. The vast area of the Arctic continental shelf was frequently emergent and covered by glaciers, resulting in massive eradications of the shelf biota [12]. Only when deglaciation started approximately 14 ka (thousand years ago) could Arctic re-colonization begin. This was accomplished by survivors that had been able to retreat into the North Atlantic or North Pacific or take refuge either in the unglaciated shelf areas of the East Siberian and Beaufort Seas or in the deeper bathyal parts of the Arctic Ocean [14,15].

Despite extensive records of Hydrozoa in the Arctic from the end of XIX century [16,17] to recent descriptions of species new to science [18–21], there is a critical gap in the species inventory, distribution records and biodiversity comparisons amongst Arctic regions [22]. For instance, Hydrozoa were not included in the first pan-Arctic inventory of macrofauna species [23], although the benthic Hydrozoa number compiled in our study is higher than for Bryozoa in shelf regions of the Arctic [23]. Data on Arctic Hydrozoa are fragmentary (mostly local surveys) and scattered (dealing only with the benthic or pelagic domain). Basic knowledge on species distribution is fundamental to biodiversity research [24], and for future efforts to follow changes in marine ecosystems connected with global warming, especially in the Arctic region.

The main aims of this study are: (1) to compile an up-to-date list of Hydrozoa occurring in Arctic waters, with their zoogeographic affinities and life cycle strategies; (2) to explore patterns of Hydrozoa distribution and diversity within the Arctic region and (3) to analyse the effect of dispersal ability on distribution. This is the first attempt to create a comprehensive Arctic species register that covers the whole class Hydrozoa, encompassing both polypoid and medusa stages and including Siphonophorae.

We address the hypothesis that the presence of a pelagic stage (holo- and meroplanktonic) is not relevant to dispersal capabilities of Hydrozoa and colonization of the Arctic.

## Methods

### Study area

**Fig 1:**
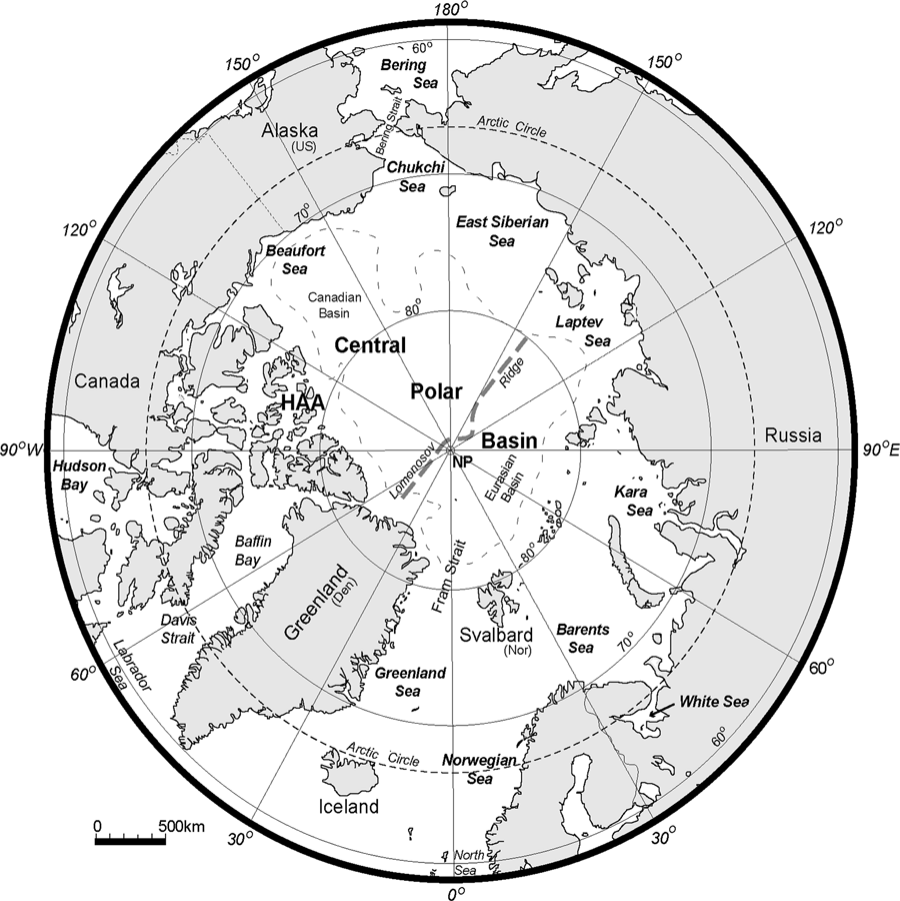
The Arctic region. An Azimuthal Equal-Area projection of the Arctic region, using the Arctic Circle (66° 33.5' N) as the boundary of the Arctic—approximately the limit of the midnight sun and polar night. All significant shelf seas are named, plus some seas that extend south of the Arctic Circle (eg. the Bering Sea). HAA identifies the Canadian High Arctic Archipelago. The Lomonosov Ridge crosses the Arctic Ocean near the North Pole (NP) and divides the Arctic's two main deep basins—the Canadian and Eurasian Basins. This aseismic ridge is 1 800km long, and rises 1 800–3 400m above the basin floor.

We use the Arctic Circle (66° 33.5' N) as the boundary of the Arctic, with some extensions. Thus, our area comprises the nearly landlocked Arctic Ocean, and adjacent shelf seas (Beaufort, Chukchi, East Siberian, Laptev, Kara, Barents Seas and the White Sea included), the Nordic Seas (Greenland and Norwegian seas with southern Greenland and Iceland included), the Labrador Sea, Baffin Bay, Hudson Bay, the High Arctic Archipelago (HAA) (Fig. 1). The Bering Sea is also incorporated because of the climate conditions and the Arctic shelf which extends through it to the Aleutian Islands. In the centre of the Arctic Ocean there are two main deep basins—the Eurasian Basin and the Canadian Basin.

Warm waters of the Atlantic and Pacific Oceans flow northward to the Arctic. Atlantic waters flow into the Arctic Ocean over the 2500 m deep, 500 km wide Fram Strait and the Nordic seas. Pacific waters flow through the shallow and narrow Bering Strait [25]. The outflow of cold waters is through the Fram Strait via the East Greenland Current and the Canadian HAA. Surface currents move counter clockwise along the Arctic coast from Atlantic to Pacific on the Eurasian side and from Pacific to Atlantic on the North American side [26].

### Data gathering and processing

An Arctic Hydrozoa list, including the zoogeographical affinity and reproductive strategy, is based on data extracted from the literature [16–21,27–87] (S1 Table). The species list was checked and rationalised for possible synonyms and the validity of each species verified with the World Register of Marine Species [88] (for list of synonyms see S2 Table). Species endemic to the Aleutian Islands and present only in Iceland were excluded from the analyses, as species occurring on the southernmost border of the study area were not good representatives of the Arctic region. The accuracy of the database extracted from historical sources may be limited by sampling biases (different sampling effort, types of gear), spatial resolution (area extension, lack of precise location information), taxonomic discrepancies (species misidentifications, miscoding of medusa and hydroid stages) and also by uneven sampling of benthic and pelagic habitats (in favour of benthic forms).

The study area was divided into regions following Spalding’s ecoregions [89] with some modifications resulting from, for example, poor representation of some regions, overlapping of bordering regions, lack of detailed coordinates of area sampled in some historical literature. We recognized the following regions: West Greenland, East Greenland, Iceland, Hudson Complex (including Hudson Bay and Strait, Foxe Basin and Ungava Bay), Beaufort Sea & High Arctic Archipelago, East Canada, Barents Sea (including Svalbard Archipelago), White Sea, Kara Sea, Laptev Sea, East Siberian Sea, Chukchi Sea, Alaska & Bering Sea, and the Central Polar Basin.

Species were classified into zoogeographical groups in relation to worldwide occurrence, as follows: 1) Arctic—noted only in high polar regions; 2) Arctic-boreal—occurring in both arctic and boreal waters; 3) boreal—found in temperate waters of the North Atlantic and North Pacific, in the present study found only in the sub-Arctic region, i.e. Iceland or/and the Bering Sea; 4) subtropical-Arctic—occurring from the Arctic to subtropical waters, with the Mediterranean region included; 5) cosmopolitan—distributed widely, and extending to tropical regions.

The species were categorised into three groups based on their life history strategy: 1) holoplanktonic species, spend their whole life as pelagic forms, 2) meroplanktonic species, have both a benthic and a pelagic stage, 3) benthic species, those that reproduce by means of fixed gonophores and those that produce short-lived, reduced medusae (eumedusoids and cryptomedusoids). For the purpose of the analyses, species that produce medusoids are grouped with benthic species because medusoids are short-lived and have limited dispersal ability (after Gibbons et al. [90]).

### Statistical analyses

Two diversity measures independent of sampling effort and sample size were employed to compare diversity at different taxonomic levels in different Arctic regions. Average taxonomic distinctness (AvTD) is the average taxonomic path length between all pairs of species [91]. Variation in taxonomic distinctness (VarTD) is the variance of the taxonomic distinctness between each pair of species about their mean value [91]. Five taxonomic levels were used in calculations: species, genus, family, order, subclass, and equal step levels between successive taxonomic levels were assumed. The master list was the species list of hydrozoan records in the Arctic (268 species). A sample data set was a species list from a particular region.

Multivariate analysis was used to identify patterns of hydroid species distribution. The similarity of species composition between Arctic regions was calculated with the Bray-Curtis index. These regions were then classified into groups by hierarchical agglomerative clustering using group-average linking, and the resulting classification presented as a dendrogram. Cluster and diversity analyses were performed with Primer package v. 6 [91].

Pearson's chi-square test was used to test for differences in the occurrence of hydrozoan species with medusa or polyp stage between the zoogeographical categories and to measure whether the proportions of the groups differed between polar regions.

## Results

### Diversity and composition

In the Arctic, the class Hydrozoa is represented by two subclasses, six orders, 54 families, 140 genera and 305 species (S1 Table, Table 1). In all, 37 species were excluded from further analyses due to uncertainty about their distribution or taxonomic status, or scarcity of worldwide records (see S1 Table, species marked with a star). Fifty two percent of the species belonged to 20 families of the order Leptothecata, and 32% to 18 families of Anthoathecata. The remaining species belonged to the orders Siphonophorae (7%), Trachymedusae (5%), Narcomedusae (3%) and Limnomedusae (1%).

**Table 1:** Species richness (N) and percentage of Hydrozoa in the Arctic and globally.

Orders	Arctic	Arctic	World	World
	N	%	N	%
Leptothecata	148	53	1795	59
Anthoathecata	79	32	961	32
Siphonophorae	19	7	166	5
Limnomedusae	2	1	33	1
Narcomedusae	7	3	36	1
Trachymedusae	13	5	50	2
Actinulida	-	-	11	0.4
Total	268	100	3052	100

Global data after Gibbons et al., 2010a (extracted from Bouillon et al., 2006), excluding all doubtful or invalid species, or synonyms. N—number of species, %—percentage of species numbers of particular order in the total number of species.

The fauna was dominated by the family Sertulariidae with 62 species (21% of the hydrozoan species known for the Arctic region). Sertulariidae was the most species-rich family in each studied Arctic region (from seven species in the Beaufort & High Arctic Archipelago (HAA) to 44 species in Alaska & Bering Sea). The next most species-rich families were Haleciidae (7%), and Campanulariidae (5%).

Highest species richness was noted in the Barents Sea, and lowest in the Hudson Complex, Beaufort Sea & HAA and Central Polar Basin (CPB) (Table 2).

**Table 2:** Number of Hydrozoa taxa (N), average taxonomic distinctness (TD) and variation in taxonomic distinctness (VarTD) in the Arctic regions.

Region	N of subclasses	N of orders	N of families	N of genera	N of species	TD	VarTD
Iceland—I	2	4	34	70	128	69.81	238.82
W Greenland—WG	2	6	35(36)	83	139	72.80	270.81
E Greenland—EG	2	6	33	63	92	73.86	245.86
Barents Sea—BS	2	6	38(39)	86	167	71.56	306.42
White Sea—WS	2	5	24(25)	49	94	68.25	281.23
Kara Sea—KS	2	6	29(30)	53	99	70.29	291.71
Laptev Sea—LS	2	6	26(27)	50	86	72.50	319.83
East Siberian Sea—ESS	2	6	25(26)	43	75	71.56	303.11
Chukchi Sea—ChS	2	6	23(24)	44	78	72.53	311.53
Alaska & Bering Sea—A&BS	2	6	39	73	149	71.33	330.37
Beaufort Sea & High Arctic Archipelago—BS&HAA	2	6	30(31)	56	57	80.98	304.81
East Canada—EC	2	5	28	53	82	72.92	321.98
Hudson Complex—HC	2	5	18	36	62	70.11	326.75
Central Polar Basin—CPB	2	6	40(41)	62	72	78.73	332.67

**Fig 2:**
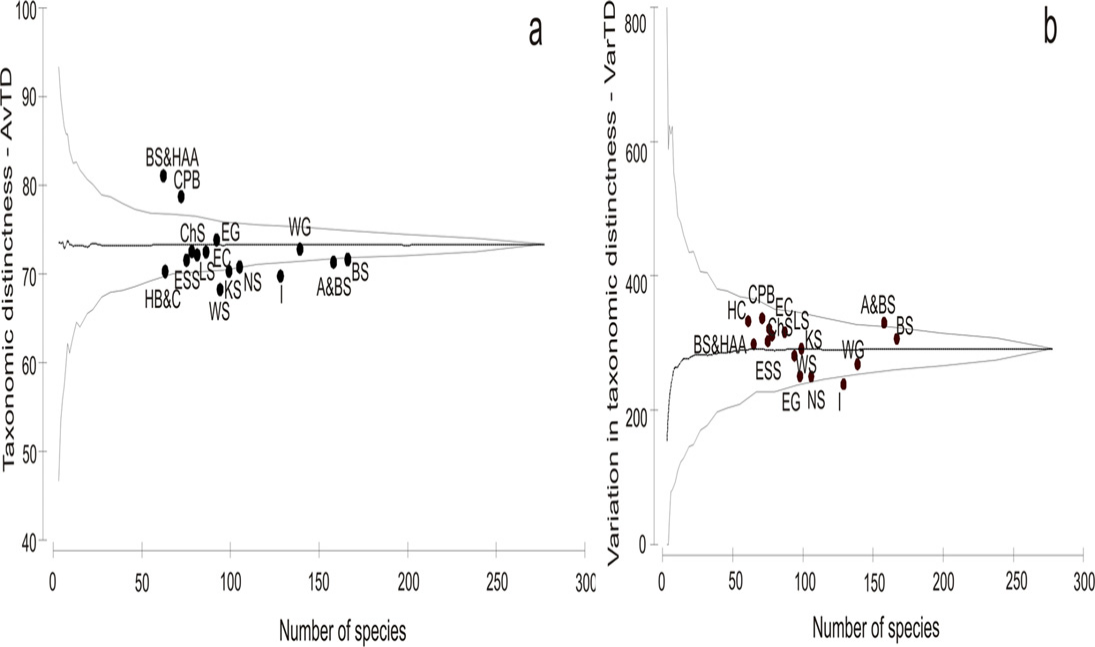
Funnel plot for simulated average taxonomic distinctness (AvTD) (a) and variation in taxonomic distinctness (VarTD) (b). Funnel plot is based on presence/absence data of Hydrozoa against observed number of species, in each Arctic region (black points). Thick line denotes AvTD for the master list. Thin lines indicate 95% probability limits for simulated AvTD. Abbreviations of regions: I—Iceland, WG—West Greenland, EG—East Greenland, BS—Barents Sea, WS—White Sea, KS—Kara Sea, LS—Laptev Sea, ESS—East Siberian Sea, CHS—Chukchi Sea, A&BS—Alaska & Bering Sea, BS&HAA—Beaufort Sea & High Arctic Archipelago, EC—East Canada, HC—Hudson Complex, CPB—Central Polar Basin.

The AvTD values for the studied regions generally fell within the 95% probability funnel. However, the value for most of the regions lay below the expected mean (Fig. 2a). Two regions had AvTD values below the expected average for the master list (White Sea and Iceland), and values for the next five regions were situated on the lower limit of the funnel (i.e., Hudson Complex, Kara, Norwegian and Barents Seas and Alaska & Bering Sea). The CPB and the Beaufort Sea & HAA, although having the lowest species richness had an AvTD significantly above expectation. In almost all regions (except Iceland) the VarTD fell within expected limits (Fig. 2b).

**Fig 3:**
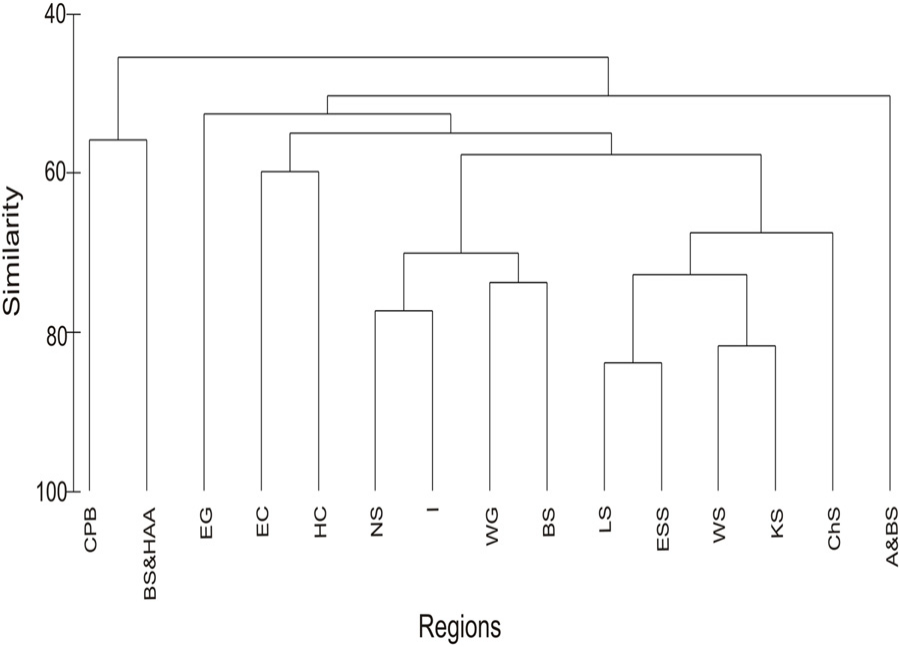
Dendrogram resulting from cluster analysis of the Bray—Curtis similarities in Arctic and subarctic water basins. Analysis based on presence/absence data of hydrozoan species list. Abbreviations of regions: I—Iceland, WG—West Greenland, EG—East Greenland, BS—Barents Sea, WS—White Sea, KS—Kara Sea, LS—Laptev Sea, ESS—East Siberian Sea, CHS—Chukchi Sea, A&BS—Alaska & Bering Sea, BS&HAA—Beaufort Sea & High Arctic Archipelago, EC—East Canada, HC—Hudson Complex, CPB—Central Polar Basin.

Cluster analysis was used to examine the similarity of species occurrence in different Arctic regions, and clustered regions into three groups. The first group comprised the CPB and the Beaufort Sea & HAA; the second group included Alaska & Bering Sea; the third group was split into four subgroups: (a) East Canada and Hudson Complex, (b) Norwegian Sea, Iceland, the Barents Sea and Western Greenland, (c) White and Kara Seas, East Siberian and Laptev Seas and Chukchi Sea, and (d) Eastern Greenland (Fig. 3).

### Zoogeographical affinities

The zoogeographical affinity of each species is presented in the S1 Table. Most species (103 species, 38%) were classified as Arctic-boreal. Nineteen per cent of species (50 species) were endemic to the Arctic region. The proportions of widely distributed taxa such as those ranging from subtropical to Arctic and cosmopolitan were 10% and 19%, respectively. Boreal representatives (34 species) constituted 13% of the total number of species.

**Fig 4:**
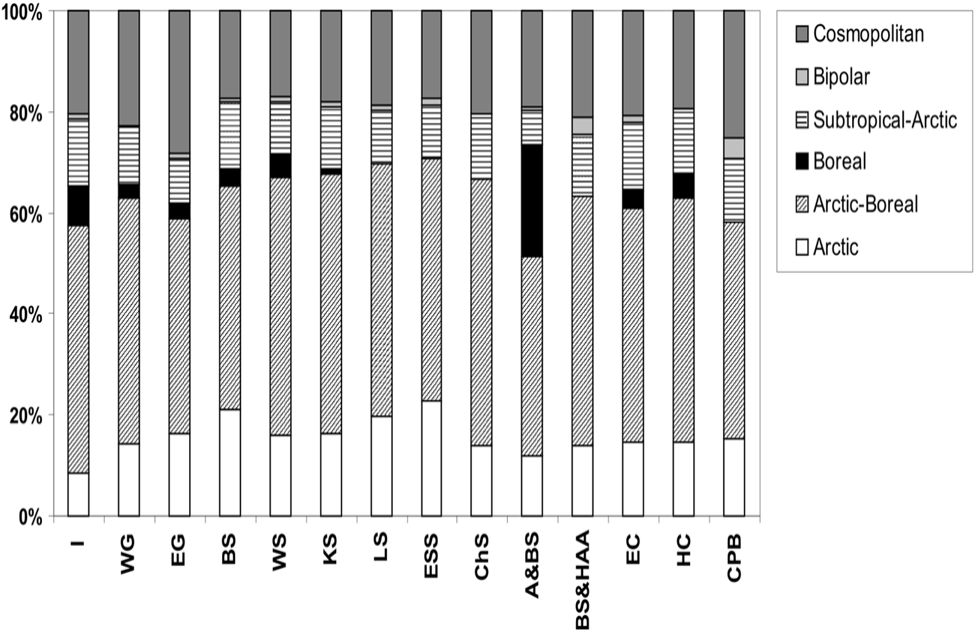
Proportion of different zoogeographical groups of Hydrozoa in the Arctic regions. Abbreviations of regions: I—Iceland, WG—West Greenland, EG—East Greenland, BS—Barents Sea, WS—White Sea, KS—Kara Sea, LS—Laptev Sea, ESS—East Siberian Sea, CHS—Chukchi Sea, A&BS—Alaska & Bering Sea, BS&HAA—Beaufort Sea & High Arctic Archipelago, EC—East Canada, HC—Hudson Complex, CPB—Central Polar Basin.

The proportions of zoogeographical groups were similar among the studied polar regions (Fig. 4). Each region was dominated by Arctic-Boreal species from 39% in Alaska & Bering Sea to 54% in the Chukchi Sea. The highest ratio of Arctic species (approximately 20%) was noted in the Barents and East Siberian Seas. Boreal species occurred in higher numbers only in Alaska & Bering Sea (22% of all species); in other regions this group reached from 0 to 8%.

### Life history strategy

Most of the species in the studied region was benthic (64%, 171 species). Twenty percent of species (54 species) displayed a holoplanktonic life history, while 16% (43 species) possessed both benthic and planktonic stages. The number of species with various life cycle strategies differed between the Arctic species pool and the global species pool (data extracted from Bouillon et al. [2] after Gibbons et al. [90]) (Pearson Chi-square goodness of fit test: χ^2^ = 10.5, df = 2, p = 0.005). While benthic species represented a similar ratio in both pools, the number of holoplanktonic species was proportionally greater in the Arctic and the number of meroplanktonic species lower.

**Fig 5:**
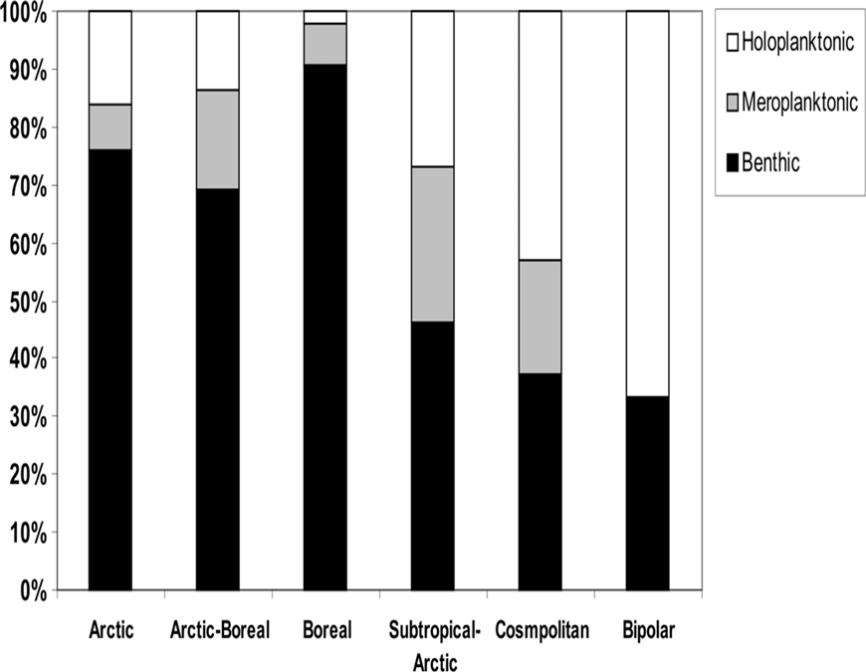
Proportion of Hydrozoa life history strategy by zoogeographical groups.

Frequency distribution of species with different life history strategies varied among zoogeographical groups (Pearson Chi-square test of independence: χ^2^ = 44.48, df = 8, p<0.001) (Fig. 5). Benthic species dominated in the Arctic, Arctic-Boreal and Boreal groups (70–90%), while species having a pelagic life stage (meroplanktonic and holoplanktonic) were more frequent in the subtropical-Arctic, cosmopolitan and bipolar groups (they comprised from 54 to 67%).

**Fig 6:**
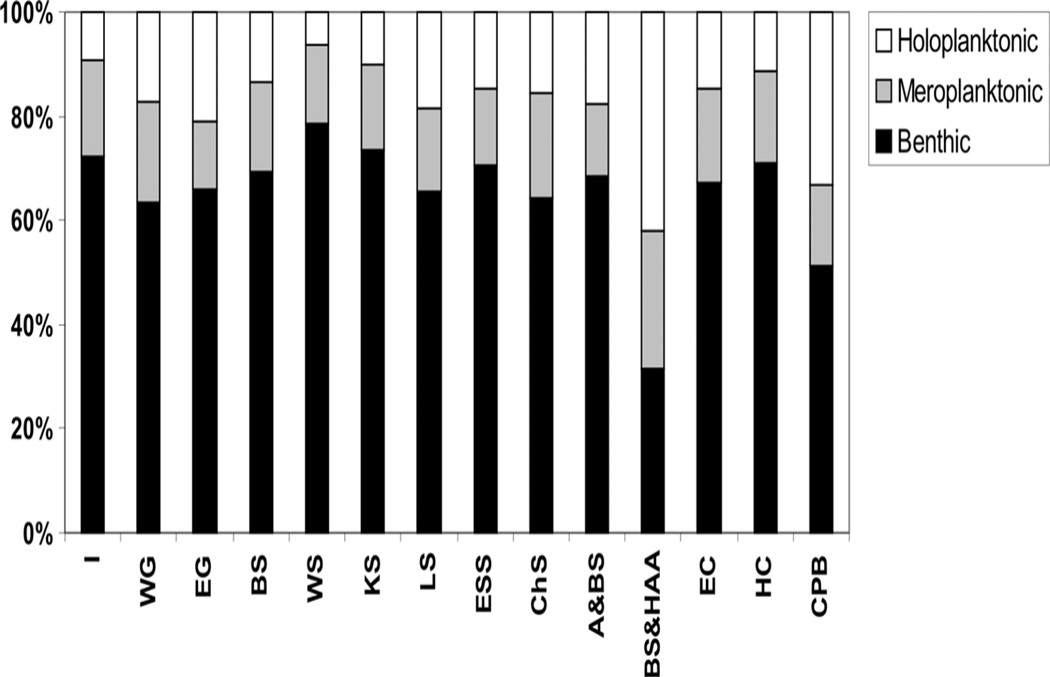
Proportion of Hydrozoa life history strategies in different Arctic regions. Abbreviations of regions: I—Iceland, WG—West Greenland, EG—East Greenland, BS—Barents Sea, WS—White Sea, KS—Kara Sea, LS—Laptev Sea, ESS—East Siberian Sea, CHS—Chukchi Sea, A&BS—Alaska & Bering Sea, BS&HAA—Beaufort Sea & High Arctic Archipelago, EC—East Canada, HC—Hudson Complex, CPB—Central Polar Basin.

The CPB and Beaufort Sea & HAA had a higher proportion of species with a pelagic stage (mostly holoplanktonic taxa) during their life history (i.e. more than 50%), compared to the other regions where, in contrary, benthic species constituted more than 60% (Fig. 6).

## Discussion

### Species richness and composition

The species list compiled from the literature revealed an overall hydrozoan species richness for the Arctic of 268. Species in the order Leptothecata dominate (55%), with Anthoathecata forming the second largest group (29%). Such proportion almost mirrors that of the global species pool, where the global ratio between these two orders is 59% to 32% [90]. Slightly higher numbers of species of Siphonophorae, Narcomedusae and Trachymedusae are observed in the Arctic region compared to the global data set, while Limnomedusae constitute 1% of hydroid species in both the Arctic and globally.

The most species rich families in the Arctic are Sertulariidae (21%) which dominated in each studied region, Haleciidae (7%) and Campanulariidae (5%). These families are the most specious in many areas in the world, including, for example, the Indian Ocean [92], Bay of Fundy [93], west coast of Australia [94], Kurile Islands [95] and Antarctica [96]. Globally, Sertulariidae is also the most specious family in the Hydrozoa (17%) [2].

Species richness (S) and other diversity indices (e.g., Shannon index) are heavily dependent on sampling effort [91]. When sampling effort is unknown or unequal (especially when it relates to historic data sets as in our case) any comparison of diversity using standard measures is problematic [91]. In addition to species richness, we used other tools (taxonomic distinctness indices) that measure biodiversity at the taxonomic level and allow comparison of species-related diversity between unbalanced samples.

Most of the Arctic regions (except the White Sea and Iceland) fall into the probability funnel for simulated average taxonomic distinctness (AvTD) and variation in taxonomic distinctness (VarTD), revealing that they involve good representatives of Arctic taxonomic diversity. The Iceland species list is not complete because boreal species that were only present in Iceland were not included in the Arctic master list (S1 Table). The species composition in Iceland can only be treated as an approximation. The White Sea sublist includes species that are most closely related, i.e. AvTD is the lowest. Ninety four of these species belong to five orders and 24 families, which means that almost every four species belong to the same family.

Some regions share patterns of taxonomic relatedness of species. For instance, Alaska & Bering Sea and the Barents Sea are characterized by high species richness and a VarTD value also above the expected level, while AvTD is at the lower limit of the funnel. Low AvTD and high VarTD values can be attributed to the relatively lower number of higher taxonomic ranks (compared to the number of species) and uneven species distribution across the hierarchical taxonomic tree.

In the Beaufort Sea & High Arctic Archipelago (HAA) and the Central Polar Basin (CPB) both AvTD and VarTD reached their highest values, even though species richness was low in these regions. This indicates that these groups of species were taxonomically very distinct (about every second species in a different family). These results confirm the independence of both taxonomic distinctness indices from the sampling effort and species richness. Increased taxonomic distinctness of assemblages has been attributed to greater stability of environmental conditions both in an evolutionary and an ecological context [97]. The Beaufort Sea & HAA and the CPB are characterized by greater overall depth compared to other shelf regions in the Arctic and successful faunistic exchange across the underwater ridges [50].

Cluster analysis was used to analyze the similarity of species occurrence in different Arctic regions. Regions separated into two main clusters: firstly, the CPB and Beaufort Sea & HAA, at 55% similarity. As mentioned above, both regions shared similar trends in diversity indices: low species richness but taxonomically very distinct (high AvTD and VarTD) and in species composition: lack of boreal species, lower percentage of benthic taxa and higher percentage of taxa having a pelagic phase (mostly holoplanktonic).

Secondly, a more diverse assemblage, consisting of three groups and two regions split off from the rest. The distinction of Alaska & Bering Sea is clearly noticeable. This region is geographically the most isolated from the Arctic Ocean. The Bering Strait is a shallow (50 m deep) and narrow (82 km wide at its narrowest point) connection between the Bering Sea and the Chukchi Sea. Thirty six species were found exclusively in Alaska & Bering Sea and did not enter the Bering Strait and Arctic Ocean. Of them, 21 species are boreal and the Bering Sea is the northernmost limit of their distribution. Fourteen species of the family Sertulariidae are absent from other Arctic regions.

E Greenland is another region separated from the remaining groups. This is due to the single occurrence of six rare species that were exclusively noted in this region. Cosmopolitan siphonophores (*Heteropyramis crystallina*, *H*. *maculata* and *Muggiaea kochi*) and the hydromedusa (*Hebella scandens*) were most likely occasional visitors. Single records of *Lafoea symmetrica* and *Hydractinia arctica* are known only from deep waters. When these species are excluded from the analysis the grouping is totally different, with E Greenland combining with W Greenland, E Canada, Iceland, the Barents and Norwegian Seas. Analyses with a presence/absence database introduce bias resulting from giving equal weight to all species, regardless of whether they are rare or common [91].

Surprisingly, the Chukchi Sea hydroid fauna is more similar to the Arctic Russian seas fauna (i.e., Laptev, East Siberian, White, Kara) than to the Alaska & Bering Sea fauna. The majority of species in the Chukchi Sea have a circumpolar distribution. Only 4 species were restricted to Pacific Arctic waters: *Earleria cellularia* and *Thuiaria cylindrica* occur only in Pacific waters, while two others *Aegina citrea* and *Nectadamas diomedeae* have a cosmopolitan distribution. The affinity of the Chukchi Sea hydroid fauna to Pacific waters is not noticeable in the species composition. This result is not in accordance with earlier studies by Dunton [14] who observed that the benthic assemblages of the Chukchi and East Siberian seas are dominated by Pacific fauna, nor those by Stepanjants [76], who noted the separation of hydroid fauna into western and eastern regions delimited by the Kara and Laptev seas.

The next group, in which the Norwegian and Barents seas, W Greenland and Iceland are clustered together, is under the direct influence of Atlantic waters, which may explain the similarity in species composition in these regions. Relatively warm and saline Atlantic water flows northward with the main inflow along the Norwegian and Barents seas and west Svalbard continental margins [98]. Another branch, a continuation of the North Atlantic Drift, flows parallel to the western coast of Greenland as the West Greenland Current [99]. Thus it is not unexpected that the fauna of W Greenland is more similar to that of the Barents Sea than, for example, the fauna of E Canada and Hudson Complex, which is under the influence of Arctic water flowing from the north. Fresh and cold Arctic water, mostly of Pacific origin, enters Baffin Bay through the HAA on the north and runs south along the shelf edge of western Baffin Bay and throughout the Hudson Complex to the Labrador Sea [99]. The majority of species found in this region have a circumpolar distribution with records noted from the Alaska & Bering Sea (85% of species in Hudson Complex and 70% in E Canada) which indicates that they are of Pacific origin colonizing these regions from the North.

### Zoogeographical affinity, life history and dispersal strategy

The hydrozoan fauna in the Arctic is dominated by Arctic-boreal species and includes a small fraction of exclusively Arctic species (19%). The dominance by Arctic-boreal forms, the lack of endemism of higher taxa and the very low level of species endemism is typical of Arctic fauna and flora [76,100,101]. The key explanation for this phenomenon might be the very short evolutionary history of the Arctic ecosystem and the fact that the Arctic is not geographically isolated [13,14,100]. The Arctic Ocean acquired its cold-water attribute (e.g., temperature drop, perennial ice cover) in the Pliocene, approximately 4 million years ago [13]. At that time, there were cycles of glacial and inter-glacial conditions with resultant ice sheet changes and global sea level variation. These events caused destruction of marine life in vast areas of the Arctic shelf biota [14]. Recolonization began relatively recently on the geological time scale. The last glaciation ended approximately 13–12 ka, enabling the present-day Arctic community to start developing. Therefore, the Arctic region is considered to be a young biota that is not yet completely established, but is still in a phase of colonization [13,14]. The species that are endemic to the Arctic and some boreal-Arctic species, most likely survived glaciations in refugia (i.e. isolated ice-free areas that retained the environmental conditions previously more widespread) [102]. Geological evidence indicates that extensive southern glacial refugia existed during the Last Glacial Maximum, 25–18 ka [15]. Lately, results of molecular studies suggest the existence of small, periglacial isolated northern ice-free areas [15], but no fossil evidence has so far been found to confirm continuous *in situ* survival of macrofauna in these areas [103]. The Chukchi, Beaufort, Laptev and East Siberian seas were unglaciated during this period but largely emergent [104,105]. Some shelf fauna may have moved southwards into the Atlantic and Pacific, retreated into unglaciated areas, or found refuge in the deeper Arctic basin [106], the latter theory being supported by the relatively large numbers of species found today within the Arctic Ocean which inhabit both shelves and continental slopes [107].

The Arctic is influenced by both Pacific and Atlantic waters, but more so by the Atlantic [26]. The connection with the Atlantic and Pacific suggests that colonization from the boreal seas could take place relatively easily unless physiological barriers (e.g., caused by differences in water temperature) are also important. These influences are reflected in the predominance of the Arctic-boreal component of the Arctic fauna which dominates each region studied. The proportion of different zoogeographical groups is similar across all regions in the Arctic, with the exception of Alaska & Bering Sea where boreal forms contributed a higher share.

Predominance of benthic species over holo- and meroplanktonic species is a common attribute of Hydrozoa worldwide. In the Arctic, 64% of species are benthic and have only a planula larva for their dispersal stage. Even in the CPB and Beaufort & HAA, benthic species constitute a high proportion of the community. The largest number of more widespread species in the Arctic (present in most ecoregions) belong to families that completely lack the medusa stage, including Sertulariidae and Haleciidae. Moreover, many cosmopolitan hydrozoans both in the Arctic (present study) and globally lack a pelagic stage [11]. Therefore, the presence of a pelagic stage (holo- or meroplanktonic) was not necessary for successful recolonization of the Arctic. However, recent molecular analyses suggest that some benthic cosmopolitan species (e.g., *Obelia geniculata*, *Lafoea dumosa*, *Nemertesia antennina*, *Plumularia setacea*) could in fact be cryptic species complexes [11,108,109). Unfortunately, Arctic specimens have not been incorporated in such analyses as yet.

The traditional view of Hydrozoa is that holo- and meroplanktonic species will have a better dispersal potential and a more extensive distribution than benthic species, whose only means of dispersal is a rather short-lived, lecithotrophic planula larva [110,111]. This idea has been derived from the general concept of a positive relationship between the length of the planktonic larval stage and geographic distribution in marine benthic invertebrates [112,113]. However, this idea has been demonstrated to be false in, for example, some gastropods [114], ascidians, scleractinian corals, most bryozoans and hydroids [115]. The successful colonization of remote habitats like Rockall island by benthic species with no planktonic larva [114] or the Azores predominantly by hydrozoans lacking a medusa phase [6], are good examples of far away colonization without a long-lived mobile stage. In most cases, it is a benthic stage that is responsible for long distance dispersal via rafting on floating objects [115]. Hydrozoans are reported to be very common rafters [6,116], with a great ability to disperse (from <100 km up to > 5000 km) on other organisms, pieces of wood, ships, and plastic items [110,116–118]. Dispersal during the benthic stage may be advantageous over long-lived larval or medusae dispersal. To establish a population in a distant area, two medusae (male and female) have to arrive at that place at the same time to mate. The probability that this may happened decreases with distance, due to the medusae’ life span and diffusion in the open ocean. Similarly, once larvae reach a distant place it must be a suitable one for settlement and metamorphosis into a hydroid colony. This colony can asexually grow and expand but in order to complete the life cycle, another colony must be present in the vicinity to provide gametes of another sex. In contrast, rafting may supply a group of individuals, probably sometimes of both sexes, which can reproduce amongst themselves. If a fertilized female colony rafts, it will brood planulae, which after settlement may establish a new population. Other means of dispersal are also known in Hydrozoa. The free hydranths may detach from a colony, travel for up to 30 days and resettle or release larvae [119,120]. Frustules or larva-like propagules produced by budding may cover some distance and themselves become reproductive [121]. If hydrozoans survived the last glaciations in northern glacial refuges (no evidence is available so far), this would also have allowed them to expand their distribution from these refuges into the nearby shelf areas with gradually retreating ice. However, this probably applies only to the most adaptable species or to species that are able to survive critical environmental stress by the formation of tolerant resting stages. Extreme physical stress causes many hydroids to transform into dormant phases, and colony regeneration follows the return of favourable conditions [122]. Different kinds of species quiescence, including dormant cysts and resting eggs, or dormant tissues in stems and stolons occur in many Arctic species (e.g. *Eudendrium album* and *Sertularia argentea* [122], *Clava multicornis* [123], *Gonothyraea loveni* [124]).

A totally different strategy is employed by holoplanktonic siphonophores, most of which are hermaphrodite, or monoecious (both sexes present on the same colony) [53] to reduce the risk of not finding a mate in a vast ocean. This may be the reason why most Siphonophora are successful cosmopolitans [125]. The majority of widely distributed hydrozoan species are mero- or holoplanktonic. Although Kramp [48] has remarked that both the medusae stage and the planula are too short lived to cross oceanic distances, pelagic stage may play a role in dispersal. Wide distribution of holopelagic hydrozoans worldwide and in the waters off South Africa has been demonstrated by Gibbons et al. [90,126].

### Limitations of the data collected

Data collected and analysed in the present study are subject to bias resulting from variation in the sampling effort undertaken through historical time in particular regions, extension of ecoregions and their accessibility. There is also a strong imbalance in the literature which underestimates the pelagic community of Hydrozoa in favour of higher sampling effort of benthic habitats; this imbalance is further exacerbated by the destructive nature of net sampling which destroys delicate jellyfish zooplankton into unrecognizable blobs. Another potential limitation of the present study (and any meta-analysis) results from integration of data through time and space when and where different people identified species in different time and regional scales. We tried to eliminate this issue by checking all possible synonyms (S2 Table).

## Conclusions

The total species richness of Hydrozoa in the Arctic was 268 with Sertulariide being the most speciose family. The hypothesis that the presence of a pelagic stage (holo- or meroplanktonic) was not necessary for successful recolonization of the Arctic is supported by our analyses. The predominance of benthic Hydrozoa suggests that the Arctic could have been colonised after the Last Glacial Maximum by hydroids rafting on floating substrata, or recolonizing from glacial refuges. Nevertheless, as holoplanktonic or meroplanktonic species outweigh the benthic species in widely distributed categories such as cosmopolitan, subtropical-Arctic and bipolar, we cannot deny that having the medusa or pelagic stage (e.g. as in the case for Siphonophora) is also an important mean of dispersal. Most Arctic hydrozoan species have Arctic-boreal distributions. The hydrozoan fauna shows a very low level of endemism, a common phenomenon in other Arctic macrofaunal groups.

We acknowledge the severe problem of imbalance in knowledge of particular regions in the Arctic as well as in sampling effort between pelagic versus benthic domains. The results of our comparative analyses may therefore change with time, when more data are available.

## Supporting Information

S1 Table List of Arctic species with their life history strategies and zoogeographical affinities. (DOC)

S2 Table Arctic species with their synonyms used in particular reference item. (DOC)
