# Supplementary material for: Trends in the Diversity, Distribution and Life History Strategy of Arctic Hydrozoa (Cnidaria)
Source: PLoS One. 2015 Mar 20;10(3):e0120204. doi: 10.1371/journal.pone.0120204 (PMC4368823; doi:10.1371/journal.pone.0120204)
Supplement: S2 Table — (DOC) [file pone.0120204.s002.doc]

**Appendix 1.** List of Arctic species with their synonyms used in particular reference item (number of item after dash). For list of references see below.

| **Species** | **Synonyms** | **References** |
| --- | --- | --- |
|  |  |  |
| **Aequoreidae** |  |  |
| *Aequorea forskalea* Péron & Lesueur, 1810 |  | 9,32 |
| **Aglaopheniidae** |  |  |
| *Aglaophenopsis bonnevieae* (Jäderholm, 1909) | *Aglaophenia compressa*-2; *Aglaophenopsis compressa*-32 | 2,32,44 |
| *Aglaophenopsis cornuta* (Fewkes, 1881) | *Aglaophenopsis cornuta*-3 | 3,44 |
| *Cladocarpus campanulatus* Ritchie, 1912 |  | 44 |
| *Cladocarpus formosus* Allman, 1877 | *Aglaophenia formosa*-2 | 2,3,20,44,53 |
| *Cladocarpus integer* (Sars, 1873) |  | 44,53 |
| *Cladocarpus pourtalesii* Verrill, 1879 |  | 53,55 |
| *Lytocarpia myriophyllum* (Linnaeus, 1758) | *Thecocarpus myriophyllum*-22 | 22,44 |
| **Bonneviellidae** |  |  |
| *Bonneviella grandis* (Allman, 1876) |  | 3,32,44,53 |
| *Bonneviella laevigata* Naumov, 1960 |  | 32 |
| *Bonneviella regia* (Nutting, 1901) |  | 32 |
| *Bonneviella superba* Nutting, 1915 |  | 32 |
| **Campanulariidae** |  |  |
| *Campanularia crenata* Allman, 1876 | *Campanularia bigena*-32; *C. speciosa*-7,12,32; *Tulpa speciosa*-53; *T. crenata*-55 | 6,12,32,44,53,55 |
| *Campanularia groenlandica* Levinsen, 1893 |  | 3,6,12,22,32,44,53 |
| *Campanularia hincksii* Alder, 1856 |  | 3,44,53 |
| *Campanularia volubilis* (Linnaeus, 1758) |  | 2,3,6,12,20,22,29,32,40,44,53,61, |
| *Clytia gracilis* (Sars, 1850) |  | 43,52,60 |
| *Clytia hemisphaerica* (Linnaeus, 1767) | *Campanularia johnstoni*-3,64 | 3,5,11,43,52,63 |
| *Clytia languida* (A. Agassiz, 1862) |  |  |
| *Gonothyraea loveni* (Allman, 1859) | *Obelia loveni*-32,53; *Laomedea hyalina*-3,20 | 3,5,15,19,31,39,43,52 |
| *Hartlaubella gelatinosa* (Pallas, 1766) | *Obelia plicata*-6; *Laomedea dichotoma*-3 |  |
| *Laomedea flexuosa* Alder, 1857 | *Obelia flexuosa*-32,53 | 3,32,53,61 |
| *Obelia dichotoma* (Linnaeus, 1758) | *Obelia plicata*-6; *Laomedea dichotoma*-3 | 3,6,44 |
| *Obelia geniculata* (Linnaeus, 1758) |  | 3,6,32,44,53,58,64 |
| *Obelia longissima* (Pallas, 1766) | *Laomedea longissima*-20,22 | 3,6,9,20,32,40,44,53,61,64,22 |
| *Orthopyxis integra* (MacGillivray, 1842) | *Campanularia integra*-3,6,12,20,22,29,32,40,53 | 3,6,12,20,22,29,32,40,44,53,61, |
| *Rhizocaulus chinensis* (Marktanner-Turneretscher, 1890) | *Verticillina chinensis*-32 | 32 |
| *Rhizocaulus verticillatus* (Linnaeus, 1758) | *Verticillina verticillata*-32; *Campanularia verticillata*-6,12,20,22,29,32 | 3,6,12,20,22,29,32,40,44,53 |
| **Campanulinidae** |  |  |
| *Calycella syringa* (Linnaeus, 1767) |  | 3,6,20,22,29,32,40,42,44,53,61 |
| *Campanulina pumila* (Clark, 1875) | *Opercularella nana*-3,32,55 | 3,32,40,44,55 |
| *Cuspidella humilis* (Alder, 1863) |  |  |
| *Cuspidella procumbens* Kramp, 1911 |  |  |
| *Cuspidella quadridentatum* Hincks, 1874 |  |  |
| *Lafoeina maxima* Levinsen, 1893 |  | 3,6,20,22,32,40,43,44,53 |
| *Lafoeina tenuis* Sars, 1874 |  | 3,29,32,44 |
| *Opercularella lacerata* (Johnson, 1847) | *Campanulina lacerata*-3,20,32,53 | 3,6,20,32,40,43,44,53 |
| **Eirenidae** |  |  |
| *Eutonina indicans* (Romanes, 1876) | *Eirine indicans*-9,32 | 1,9,32,24,44 |
| **Haleciidae** |  |  |
| *Halecium* *arcticum* Ronowicz & Schuchert, 2007 | *H. undulatum*-6 | 6,41 |
| *Halecium beanii* (Johnston, 1838) |  | 3,29,32,43,44,53 |
| *Halecium birulai* Spassky, 1929 |  | 32,44,53 |
| *Halecium corrugatum* Nutting, 1899 |  | 3,32,53,55 |
| *Halecium curvicaule* Lorenz,1886 | *H. kukenthali*-29 | 3,6,20,22,29,32,40,43,44,53,55 |
| *Halecium groenladicum* Kramp, 1911 |  | 6,20,22,32,44,53 |
| *Halecium halecinum* (Linnaeus, 1758) |  | 3,22,29,32,53,61 |
| *Halecium harrimani* Nutting, 1901 |  | 33 |
| *Halecium irregulare* Bonnevie, 1899 |  |  |
| *Halecium laeve* Kramp, 1932 |  | 20,44 |
| *Halecium labrosum* Alder, 1859 |  | 3,6,20,22,29,32,44,53,61 |
| *Halecium marsupiale* Bergh, 1887 |  | 32,53 |
| *Halecium minutum* Broch, 1903 |  | 3,6,20,22,40,43,44 |
| *Halecium mirabile* Schydlowsky, 1902 |  | 3,32,40,43 |
| *Halecium muricatum* (Ellis & Solander, 1786) |  | 2,3,6,16,20,22,32,33,40,43,44,53 |
| *Halecium ornatum* Nutting, 1901 |  | 3,33 |
| *Halecium reversum* Nutting, 1901 |  | 32,33,53 |
| *Halecium scutum* Clark, 1877 | *H. beringi*-32 | 3,6,32,33,55 |
| *Halecium speciosum* Nutting, 1901 |  | 6,32,33,40,43,53 |
| *Halecium tenellum* Hincks,1861 |  | 3,20,22,32,44,53,61 |
| *Halecium textum* Kramp, 1911 |  | 40,43,44 |
| *Halecium undulatum* Billard, 1921 |  |  |
| **Halopterididae** |  |  |
| *Nuditheca dalli* (Clark, 1876) | *Macrorhynchia dalii*-8 | 8,32 |
| *Nuditheca tetrandra* Naumov, 1960 |  | 32 |
| *Schizotricha polaris* Naumov, 1960 |  | 32,53 |
| *Schizotricha variabilis* Bonnevie, 1899 | *Plumularia variabilis*-2 | 2,32,44,53 |
| **Hebellidae** |  |  |
| *Hebella scandens* (Bale, 1888) | *H. calcarata*-22 | 22 |
| **Kirchenpaueriidae** |  |  |
| *Kirchenpaueria fragilis* (Hamann, 1882) | *Plumularia fragilis*-2,32,53 | 2,32,53,55 |
| *Kirchenpaueria plumularioides* (Clark, 1877) | *Halecium plumularioides*-8 | 8 |
| **Lafoeidae** |  |  |
| *Acryptolaria conferta* (Allman, 1877) | *Cryptolaria profunda*-32 | 32,44 |
| *Acryptolaria flabellum* (Allman, 1888) | *Cryptolaria flabellum*-32 | 32 |
| *Filellum serpens* (Hassall, 1848) |  | 3,6,20,22,32,40,44,53,61 |
| *Grammaria abietina* (M. Sars, 1850) | *Lafoea abietina*-2 | 2,3,6,20,22,44,53,61 |
| *Grammaria borealis* (Levinsen, 1893) | *Cryptolaria borealis*-6,32; *Acryptolaria borealis*-53 | 3,6,20,32,44,53 |
| *Grammaria immersa* Nutting, 1901 |  | 3,6,22,32,44,53 |
| *Halisiphonia arctica* Kramp, 1932 |  | 44 |
| *Lafoea dumosa* (Fleming, 1820) | *L. gracillima*-6,20,29; *L. pocillum*-8,32,53; *L. fruticosa*-2,3,6,32 | 2,3,6,8,20,29,32,43,44,53,61 |
| *Lafoea grandis* Hincks, 1874 |  | 32,53 |
| *Lafoea symmetrica* Bonnevie, 1899 |  | 2 |
| Zygophylax pinnata (Sars, 1873) |  | 53,55 |
| **Laodiceidae** |  |  |
| *Ptychogena hyperborea* Kramp, 1942 |  | 19,25,39 |
| *Ptychogena lactea* Agassiz, 1865 |  | 6,9,13,24,32,50,53,54,55,58,63,64 |
| *Staurostoma mertensii* (Brandt, 1834) | *Staurophora mertensi*-50,58,59,63; *Cuspidella mertensii*-32 | 32,50,53,55,58,59,63,64 |
| **Lovenellidae** |  |  |
| *Eucheilota ventricularis* McCrady, 1859 |  | 25 |
| *Lovenella producta* (G.O. Sars, 1874) | *Campanulina producta*-3 | 3,44 |
| **Melicertidae** |  |  |
| *Melicertum octocostatum* M.Sars,1835 | *Melicertum campanula*-9,32,64 | 9,14,24,25,32,50,53,54,58,63,64 |
| **Mitrocomidae** |  |  |
| *Cosmetira pilosella* Forbes, 1848 |  |  |
| *Earleria cellularia* (A.Agassiz, 1862) | *Halistaura cellularia*-24,50 | 24,25,50 |
| *Halopsis ocellata* Agassiz, 1863 |  | 21,25,32,53,63,64 |
| *Mitrocomella polydiademata* (Romanes, 1876) | *Cuspidella grandis*-32; *C. polydiademata*-32,64 | 25,32,53,54,55,62,63,64 |
| **Phialellidae** |  |  |
| *Phialella quadrata* (Forbes, 1848) |  | 61 |
| **Plumulariidae** |  |  |
| *Nemertesia antennina* (Linnaeus, 1758) | *Antennularia antennina*-3 | 3,6,20,32,44,53 |
| *Polyplumaria gracillima* (Sars, 1873) | *Polynemertesia gracillima*-32,53 | 22,32,44,53 |
| **Sertulariidae** |  |  |
| *Abietinaria abietina* (Linnaeus, 1758) | *Diphasia abietina*-3,20 | 3,6,20,22,32,44,53,55 |
| *Abietinaria compressa* (Merezhkovskii, 1878) |  | 32 |
| *Abietinaria filicula* (Ellis & Solander, 1786) | *Diphasia abitina* var. *filicula*-3; *Sertularia filicula*-8 | 3,6,8,32,44,53 |
| *Abietinaria fusca* (Johnston, 1847) |  | 32,44,53 |
| *Abietinaria gigantea* (Clark, 1877) | *Thuiaria gigantea*-8 | 8,32 |
| *Abietinaria gracilis* Nutting, 1904 |  | 31 |
| *Abietinaria inconstans* (Clark, 1877) | *Thuiaria costata*-33 | 32,33 |
| *Abietinaria kincaidi* (Nutting, 1901) |  | 32 |
| *Abietinaria pulchra* (Nutting, 1904) | *Diphasia pulchra*-6,20 | 6,20,32,40,43,44,53 |
| *Abietinaria thuiarioides* (Clark, 1877) | *Sertularia thuiarioides*-8 | 8,32,44 |
| *Abietinaria turgida* (Clark, 1877) | *Thuiaria turgida*-8 | 6,8,32,53,55 |
| *Abietinaria variabilis* (Clark, 1877) | *Diphasia variabilis*-3; *Sertularia variabilis*-8 | 3,8,32 |
| *Diphasia attenuata* (Hincks, 1866) |  | 44 |
| *Diphasia fallax* (Johnston, 1847) | *Dynamena fala*-2 | 2,3,22,32,44,53 |
| *Diphasia rosacea* (Linnaeus, 1758) |  | 3,6,32,44,53 |
| *Dynamena pumila* (Linnaeus, 1758) | *Sertularia pumila*-3 | 3,6,32,44,53 |
| *Hydrallmania falcata* (Linnaeus, 1758) |  | 2,3,22,32,44,53 |
| *Sertularella albida* Kirchenpauer, 1884 |  | 32 |
| *Sertularella complexa* Nutting, 1904 |  | 32 |
| *Sertularella erratum* Vervoort & Watson, 2003 | *Sertularella reticulata*-32 | 32 |
| *Sertularella fusiformis* (Hincks, 1861) | *Sertularella pellucida*-32,53 | 6,22,32,53 |
| *Sertularella gayi* (Lamouroux, 1821) |  | 3,44 |
| *Sertularella gigantea* Mereschowsky, 1878 |  | 3,20,22,32,53,55 |
| *Sertularella pinnata* Clark, 1876 (?) |  | 3,6,32 |
| *Sertularella polyzonias* (Linnaeus, 1758) | *S. polyzonias* var. *gigantea*-3,20,22 | 2,3,6,8,20,22,32,33,43,44 |
| *Sertularella rugosa* (Linnaeus, 1758) |  | 3,8,32,40,43,44,53 |
| *Sertularella tenella* (Alder, 1856) |  | 3,6,22,32,40,43,44,53 |
| *Sertularia albimaris* Mereschowsky, 1878 |  | 3,32,40,43,53,55 |
| *Sertularia argentea* Linnaeus, 1758 | *S. cupressina*-6,32,53 | 6,32,40,43,44,53 |
| *Sertularia converrucosa* Naumov, 1960 |  | 32 |
| *Sertularia cupressoides* Clark, 1876 |  | 8,32,33,40,43,53,55 |
| *Sertularia fabricii* Levinsen, 1893 |  | 3,6,20,43,44 |
| *Sertularia mirabilis* (Verrill, 1873) | *Diphasia mirabilis*-8 | 3,6,8,20,32,40,43,44,53 |
| *Sertularia plumosa* (Clark, 1876) | *Thuiaria plumosa*-8 | 3,6,8,32,53,55 |
| *Sertularia robusta* (Clark, 1876) | *Thuiaria robusta*-8 | 3,6,8,32 |
| *Sertularia schmidti* Kudelin, 1914 |  | 6,32,40,43,44 |
| *Sertularia similis* Clark, 1877 |  | 6,8,32,40,43,44,53 |
| *Sertularia tenera*  Sars, 1874 |  | 3,6,20,22,29,32,44,53 |
| *Sertularia tolli* (Jäderholm, 1908) |  | 32,53 |
| *Symplectoscyphus pinnatus* (Clark, 1876) |  | 32,55 |
| *Symplectoscyphus tricuspidatus* (Alder, 1856) | *Sertularella tricuspidata*-2,6,20,22,32,53 | 2,6,20,22,29,32,40,43,44,53,61 |
| *Tamarisca tamarisca* (Linnaeus, 1758) | *Sertularella tamarisca*-3; *Sertomma tamarisca*-20 | 3,32,43,44,53,55 |
| *Thuiaria alternitheca* Levinsen, 1893 |  | 3,6,20,44,53 |
| *Thuiaria arctica* (Bonnevie, 1899) | *Saleginopsis arctica*-2 | 2,3,32,44,53 |
| *Thuiaria articulata* (Pallas, 1766) | *T. barentsi*-32; *T. lonchitis*-6,53 | 2,6,32,40,43,44,53 |
| *Thuiaria breitfussi* (Kudelin, 1914) |  | 32,53,55 |
| *Thuiaria carica* Levinsen, 1893 | *T. kirchenpaueri*-29 | 3,6,29,32,44,53 |
| *Thuiaria cedrina* (Linnaeus, 1758) |  | 32 |
| *Thuiaria cornigera* Kudelin, 1914 |  | 32 |
| *Thuiaria cupressoides* (Lepechin, 1783) |  | 32,53 |
| *Thuiaria cylindrica* Clark, 1876 |  | 3,8,32,53,55 |
| *Thuiaria decemserialis* (Merezhkovskii, 1878) |  | 3,32,53 |
| *Thuiaria hartlaubi* (Nutting, 1904) |  | 32,44 |
| *Thuiaria hippuris* Allman, 1874 |  | 22 |
| *Thuiaria kudelini* Naumov, 1960 |  | 32 |
| *Thuiaria laxa* Allman, 1874 |  | 3,6,22,32,40,43,44,53 |
| *Thuiaria mereschkowskii* Kudelin, 1914 |  | 32 |
| *Thuiaria obsoleta* (Lepechin, 1781) | *Saleginopsis obsoleta*-2 | 2,3,32,53 |
| *Thuiaria pinaster* (Lepechin, 1783) |  | 3 |
| *Thuiaria sachalini* Kudelin, 1914 |  | 44 |
| *Thuiaria uschakovi* Naumov, 1960 |  | 32,54 |
| *Thuiaria thuja* (Linnaeus, 1758) |  | 3,6,20,22,32,44,53 |
| **Tiarannidae** |  |  |
| *Chromatonema rubrum* Fewkes, 1882 |  | 58 |
| *Modeeria rotunda* (Quoy & Gaimard, 1827) | *Stegopoma fastigiatum*-3,20,32 | 3,20,32,53 |
| *Stegopoma plicatile* (M. Sars, 1863) | *Modeeria plicatile*-53 | 3,6,22,32,40,44,53,55 |
| **Tiaropsidae** |  |  |
| *Tiaropsis multicirrata* (Sars, 1835) |  | 10,13,14,24,25,32,50,53,54,55,58,63 |
| **Order: Anthoathecata** |  |  |
| **Suborder: Capitata** |  |  |
| **Acaulidae** |  |  |
| *Acaulis primarius* Stimpson, 1854 |  | 22,32,44,55 |
| **Boreohydridae** |  |  |
| *Boreohydra simplex* Westblad, 1937 |  | 44,45 |
| **Candelabridae** |  |  |
| *Candelabrum phrygium* (Fabricius, 1780) | *Myriothela phrygia*-2,3,7,32 | 2,3,7,20,32,44,45,53,61 |
| *Candelabrum verrucosum* (Bonnevie, 1989) |  | 2,45 |
| *Monocoryne gigantea* (Bonnevie, 1898) |  | 7,45,53,55 |
| **Corymorphidae** |  |  |
| *Branchiocerianthus reniformis* Broch, 1918 |  | 44 |
| *Corymorpha carnea* (Clark, 1876) | *Rhizonema carnea*-8 | 8 |
| *Corymorpha glacialis* Sars, 1859 | *Monocaulus glacialis*-55 | 3,32,44,53,55 |
| *Corymorpha groenlandica* (Allman, 1876) | *Monocaulus groenlandica*-55; *Lampra purpurea*-2 | 2,3,7,32,44,48,53,55 |
| *Corymorpha nana* Alder, 1857 |  | 2,48,49 |
| *Corymorpha nutans* M. Sars, 1835 |  | 3,16,44,48,49 |
| *Corymorpha typica* (Uchida,1927) | Gotoea typica-51 | 51 |
| *Euphysa aurata* Forbes, 1848 | *Corymorpha aurata*-32,64 | 25,32,44,48,53,55,58,64 |
| *Euphysa flammea* (Linko, 1905) | *Corymorpha flammea*-9,32,64 | 9,10,13,14,24,32,50,53,55,58,59,62,63,64 |
| *Euphysa tentaculata* Linko, 1905 | *Corymorpha tentaculata*-32,64 | 1,21,32,48,53,58,64 |
| *Gymnogonos crassicornis* Bonnevie, 1898 |  | 42,43,44,48,53,57 |
| *Gymnogonos obvolutus* Kramp, 1933 | *Corymorpha obvoluta*-22 | 22,48,53,57 |
| *Paragotoea bathybia* Kramp, 1942 | *Paragotoea elegans*-18 | 18,19,39,48,53,57 |
| **Corynidae** |  |  |
| *Coryne hincksi* Bonnevie, 1898 |  | 7,44 |
| *Coryne pusilla* (Gaertner, 1774) |  |  |
| *Sarsia brachygaster* Grönberg, 1898 sp. inq. |  |  |
| *Sarsia lovenii* (M. Sars, 1846) | *Coryne lovenii*-32,53 | 22,32,40,43,44,53 |
| *Sarsia princeps* (Haeckel, 1879) | *Coryne princeps*-32; *Coryne principes*-9 | 9,10,13,14,18,24,32,50,53,55,58,63,64 |
| *Sarsia tubulosa* (M. Sars, 1835) | *Coryne tubulosa*-9,32,64; *C. sarsi*-20; *S. barentsi*-58 | 1,7,9,10,20,24,25,32,44,53,58,61,63,64 |
| *Stauridiosarsia gemmifera* (Forbes, 1848) | *Sarsia gemmifera*-64 | 64 |
| *Stauridiosarsia producta* (Wright, 1858) |  | 32,44,55 |
| **Tubulariidae** |  |  |
| *Bouillonia cornucopia* (Bonnevie, 1898) | *Tubularia cornucopia*-2 | 2,44,48,53 |
| *Ectopleura larynx* (Ellis & Solander, 1786) | *Tubularia larynx*-2,4,32,55 | 2,3,32,44,53,55 |
| *Hybocodon prolifer* Agassiz, 1860 | *Tubularia prolifer*-2,9,32,53,64; *H. christinae*-63 | 1,2,9,10,24,32,44,48,50,53,54,55,58,63,64 |
| *Tubularia indivisa* Linnaeus, 1758 | *Tubularia simplex*-32,55 | 3,8,32,44,53,55 |
| *Tubularia regalis* Boeck, 1860 | *T. variabilis*-2 | 2,3,7,8,20,34,44,53,54 |
| *Zyzzyzus robustus* Petersen, 1990 |  | 34 |
| **Protohydridae** |  |  |
| *Protohydra leuckarti* Greeff, 1870 |  | 53,55 |
| *Sympagohydra tuuli* Piraino et al. 2008 |  | 35 |
| **Capitata incertae sedis** |  |  |
| *Plotocnide borealis* Wagner, 1885 | *Eucodonium arctica*-15,50 | 15,18,19,48,50,51,53,54,55,58,59,63,64 |
| *Rhabdoon reesi* (Shirley & Leung, 1970) | *Yakovia polinae-*17,18,56*;Pararhysomedusa reesi*-51 | 17,18,19,39,48,51,53,56 |
| **Suborder: Filifera** |  |  |
| **Bougainvilliidae** |  |  |
| *Bougainvillia principis* (Steenstrup, 1850) |  | 24,32,53,54,58,63,64 |
| *Bougainvillia superciliaris* (L. Agassiz, 1849) |  | 9,10,15,24,42,43,50,53,54,55,58,62,63,64 |
| *Chiarella centripetalis* Maas, 1897 | *Rathkea jaschnowi*-9,32; *Ch. jaschnowi*-24 | 9,24,32 |
| *Dicoryne conferta* (Alder, 1856) |  | 3,44,53 |
| *Garveia polarsterni* Stepanjants, 2001 |  | 53,55 |
| *Rhizorhagium roseum* Sars, 1874 | *Perigonimus roseus*-32 | 3,7,32,40,42,43,44,53 |
| **Bythotiaridae** |  |  |
| *Bythotiara depressa* Naumov, 1960 |  | 32 |
| *Calycopsis birulai* (Linko, 1913) | *Eumedusa birulai*-13,18,50 | 13,14,18,32,50,53,54,55,63,64 |
| *Calycopsis nematomorpha Bigelow, 1913* | *C. nematophora*-9,32 | 1,9,24,32,58 |
| *Meator rubatra* Bigelow, 1913 |  | 24 |
| **Eudendriidae** |  |  |
| *Eudendrium album* Nutting, 1898 | *E. islandicum*-40 | 40,42,43,44,47 |
| *Eudendrium annulatum* Norman, 1864 |  | 3,20,32,40,42,43,44,47,53 |
| [*Eudendrium arbuscula* Wright, 1859](http://www.marbef.org/data/aphia.php?p=taxdetails&id=117541) |  | 42,43 |
| *Eudendrium capillare* Alder, 1856 |  | 3,7,20,29,32,42,43,44,47,53 |
| *Eudendrium rameum* (Pallas, 1766) |  | 2,3,7,22,29,32,40,42,43,44,47,53 |
| *Eudendrium ramosum* (Linnaeus, 1758) |  | 3,22,32,40,43,44,47,53,55 |
| *Eudendrium tenellum* Allman, 1877 sp.inq. |  |  |
| *Eudendrium unispirum* Schuchert, 2008 |  | 42,43,47,49 |
| *Eudendrium vaginatum* Allman, 1863 |  | 12,42,43,47,49,61 |
| **Hydractiniidae** |  |  |
| *Clava multicornis* (Forsskål, 1775) | *C. squamata*-3,22 | 3,22,32,42,43,44,53 |
| *Clavactinia serrata* (Kramp, 1943) | *Hydractinia serrata*-22,32,42,43,46,53,54 | 22,32,42,43,44,46,53,54 |
| *Hydractinia arctica* (Jäderholm, 1902) |  | 44,46 |
| *Hydractinia carica* Bergh, 1887 | *H. minuta*-2 | 2,7,12,32,43,44,46,53,55,61 |
| *Hydractinia echinata* (Fleming, 1828) |  | 3,12,32,44,46,53,55 |
| *Hydractinia ingolfi* Kramp, 1932 |  | 20,44,46 |
| *Hydractinia monocarpa* Allman, 1876 |  | 3,7,29,32,44,46,53 |
| *Hydractinia* cf. *monoon* (Hirohito, 1988) |  | 42 |
| *Hydractinia sarsii* Steenstrup, 1850 |  | 2,44,46 |
| *Podocoryna borealis* (Mayer, 1900) |  | 3,44 |
| *Podocoryna carnea* (M. Sars, 1846) |  | 3,44,53,58 |
| *Schuchertinia allmani* (Bonnevie, 1898) | *Hydractinia allmani*-2,20,32,44,46,53 | 2,3,22,32,44,46,53,55 |
| *Schuchertinia antonii* (Miglietta, 2006) |  | 30 |
| **Margelopsidae** |  |  |
| *Margelopsis hartlaubi* Browne, 1903 |  | 19 |
| **Oceaniidae** |  |  |
| *Rhizogeton nudus* Broch, 1910 | *R. nudum*-3,53 | 3,7,20,22,42,43,44,53,61 |
| *Similomerona nematophora* (Antsulevich, 1986) | *Rhizogeton nematophorum*-53 | 42,43,53,55 |
| **Pandeidae** |  |  |
| *Catablema multicirratum* Kishinouye, 1910 | *C. multicirrata*-59 | 24,25,58,59 |
| *Catablema vesicarium* (A. Agassiz, 1862) | *C. vesicaria*-53 | 10,18,24,25,49,50,53,54,55,58,62,63 |
| *Halitholus cirratus* Hartlaub, 1913 |  | 10,13,14,25,49,50,54,58,61,63 |
| *Halitholus pauper* Hartlaub, 1913 |  | 10,25,50,58 |
| *Halitholus yoldiaarcticae* (Birula, 1897) sp. inq. | Perigonimus yoldia-arcticae-3,9,32,64 | 3,9,18,22,32,43,44,53,55,64 |
| *Leuckartiara nobilis* Hartlaub, 1914 |  | 15,24,25,50 |
| *Neoturris abyssi* (G.O. Sars 1874) | *Perigonimus abbysi*-3,32,64,2,22 | 2,3,22,32,64 |
| *Neoturris breviconis* (Murbach & Shearer, 1902) | *Perigonimus brevicornis*-9,32; *Leucartiara breviconis*-15,24,50,58 | 9,10,15,24,25,32,50,58 |
| **Protiaridae** |  |  |
| *Paratiara digitalis* Kramp & Damus, 1925 |  | 54 |
| **Rathkeidae** |  |  |
| *Rathkea octopunctata* (M. Sars, 1835) |  | 1,10,15,24,25,50,53,54,58,62,63,64 |
| **Stylasteridae** |  |  |
| *Crypthelia trophostega* Fisher, 1938 |  | 5,11,32 |
| *Cyclohelia lamellata* Cairns, 1991 |  | 5 |
| *Distichopora borealis* Fisher, 1938 |  | 5 |
| *Errinopora dichotoma* Cairns & Lindner, 2011 |  | 5 |
| *Errinopora disticha* Cairns & Lindner, 2011 |  | 5 |
| *Errinopora fisheri* Cairns & Lindner, 2011 |  | 5 |
| *Errinopora nanneca* Fisher, 1938 |  | 3,11 |
| *Errinopora undulata* Cairns & Lindner, 2011 |  | 5 |
| *Errinopora zarhyncha* Fisher, 1938 |  | 3,11 |
| *Stylaster alaskanus* Fisher, 1938 |  | 11 |
| *Stylaster brochi* (Fisher, 1938) | *Allopora brochi*-11,32 | 3,5,11,32 |
| *Stylaster campylecus* (Fisher, 1938) | *Allopora polyorchis*-11,32 | 3,11,32 |
| *Stylaster elassotomus* (Fisher, 1938) |  | 11 |
| *Stylaster erubescens groenlandicus* Zibrowius & Cairns, 1992 |  | 46 |
| *Stylaster gemmascens* (Esper, 1794) |  | 4,32,46 |
| *Stylaster leptostylus* (Fisher, 1938) |  |  |
| *Stylaster parageus parageus* (Fisher, 1938) |  | 3 |
| *Stylaster repandus* Cairns & Lindner, 2011 |  | 5 |
| *Stylaster roseus* (Pallas, 1766) |  | 5 |
| *Stylaster stejnegeri* (Fisher, 1938) | *Allopora stejnegeri*-11 | 11 |
| *Stylaster trachystomus* (Fisher, 1938) |  | 11 |
| *Stylaster verrilli* (Dall, 1884) | *Allopora verrilli*-32 | 3,32 |
| **Zancleidae** |  |  |
| *Zanclea* sp. |  |  |
| **Order: Siphonophorae** |  |  |
| **Clausophyidae** |  |  |
| *Chuniphyes moserae* Totton, 1954 |  | 31,52 |
| *Chuniphyes multidentata* Lens & van Riemsdijk, 1908 |  | 21,60 |
| *Crystallophyes amygdalina* (Moser, 1925) |  | 27,59,60 |
| *Heteropyramis crystallina* (Moser, 1925) | *Thalassophyes crystallina-*60 | 60 |
| *Heteropyramis maculata* Moser, 1925 |  | 27 |
| **Diphyidae** |  |  |
| *Dimophyes arctica* (Chun, 1897) |  | 9,10,13,17,21,26,27,28,31,37,50,51,52,53,60,64 |
| *Gilia reticulata* (Totton, 1954) | *Lensia reticulata-*27,31 | 27,31,36,52 |
| *Lensia achilles* Totton, 1941 |  | 27,31,52 |
| *Lensia conoidea* (Keferstein & Ehlers, 1860) |  | 21,27,60 |
| *Muggiaea bargmannae* Totton, 1954 |  | 14,17,27,28,31,39,53,59 |
| *Muggiaea kochi* (Will, 1844) |  | 60 |
| **Hippopodiidae** |  |  |
| *Vogtia serrata* |  | 9,21,31,52 |
| **Pyrostephidae** |  |  |
| *Bargmannia* sp. |  |  |
| **Prayidae** |  |  |
| *Nectadamas diomedeae* (Bigelow, 1911) | *Nectopyramis diomedea*-51,53 | 51,52,53 |
| *Rosacea plicata* Bigelow, 1911 |  |  |
| **Agalmatidae** |  |  |
| *Agalma okeni* Eschscholtz, 1825 |  | 51 |
| *Nanomia cara* Agassiz, 1865 | *Stephanomia cara*-21 | 21,27,60 |
| *Marrus orthocanna* (Kramp, 1942) | *Stephanomia orthocanna*-21; *M. antarcticus pacifica*-52 | 21,26,27,28,39,52,53,64 |
| *Rudjakovia plicata* Margulis, 1982 |  | 28,53,54 |
| **Apolemiidae** |  |  |
| *Apolemia vitiazi* (Stepanjants, 1967) | *Ramosia vitiazi*-31,52; *Ramosa vitiazi*-9 | 9,31,52 |
| **Physophoridae** |  |  |
| *Physophora hydrostatica* Forskål, 1775 |  | 21,53,60,64 |
| **Order: Trachymedusae** |  |  |
| **Ptychogastriidae** |  |  |
| *Ptychogastria polaris* Allman, 1878 |  | 17,19,25,32,39,50,53,54,55,58,64 |
| **Halicreatidae** |  |  |
| *Botrynema brucei* Browne, 1908 |  | 9,17,19,25,39,51,55 |
| *Botrynema ellinorae* (Hartlaub, 1909) |  | 17,18,19,25,37,39,51,55,58 |
| *Halicreas minimum* Fewkes, 1882 | *H. papiliosum*-58 | 9,25,32,58,64 |
| *Haliscera bigelowi* Kramp, 1947 |  | 32,53 |
| *Homoeonema platygonon* Browne, 1903 |  | 17,18,19,25,32,37,51,53,54,55,58,63,64 |
| **Rhopalonematidae** |  |  |
| *Aglantha digitale* (O.F. Müller, 1776) |  | 9,10,13,14,17,18,23,25,37,39,50,51,53,54,55,58,59,63,64 |
| *Benthocodon hyalinus* Larson & Harbison, 1990 |  | 19 |
| *Crossota brunnea* Vanhöffen, 1902 |  | 9 |
| *Crossota millsae* Thuesen, 2003 |  | 19,39 |
| *Crossota norvegica* Vanhöffen, 1902 |  | 19,39 |
| *Crossota rufobrunnea* (Kramp, 1913) |  | 1,17,23,39,55,58 |
| *Pantachogon haeckeli* Maas, 1893 |  | 1,9,23,24,25,32,50,54,58,63,64 |
| *Sminthea arctica* (Hartlaub, 1909) |  | 17,19,25,37,39,51,58,59 |
| **Order: Limnomedusae** |  |  |
| **Monobrachiidae** |  |  |
| *Monobrachium parasitum* Mereschkowsky, 1877 |  | 2,3,32,44,53 |
| **Olindiidae** |  |  |
| *Eperetmus typus* Bigelow, 1915 |  | 25,58 |
| *Gonionemus vertens* A. Agassiz, 1862 |  | 25 |
| **Order: Narcomedusae** |  |  |
| **Aeginidae** |  |  |
| *Aegina citrea* Eschscholtz, 1829 | *Aegina rosea*-9,32,50,58 | 9,32,50,58 |
| *Aeginopsis laurentii* Brandt, 1835 |  | 10,13,14,15,17,18,25,50,51,53,54,55,58,59,64 |
| *Aeginura grimaldii* Maas, 1904 |  | 24,25,32,58 |
| *Bathykorus bouilloni* Rascoff, 2010 |  | 19,38 |
| *Solmundella bitentaculata* (Quoy & Gaimard, 1833) |  | 19,32,39,51 |
| **Cuninidae** |  |  |
| *Solmissus incisa* (Fewkes, 1886) |  | 24,25,58 |
| **Solmarisidae** |  |  |
| *Solmaris corona* (Keferstein & Ehlers, 1861) |  | 24,64 |

References

1. Arai MN, Brinkmann-Voss A (1980) Hydromedusae of British Columbia and Puget Sound. Canadian Bulletin of Fisheries and Aquatic Sciences 204: 1-192.
2. Bonnevie K (1899) Hydroida. The Norwegian North-Atlantic Expedition 1876-1878 7: 1-103.
3. Broch H (1909) Die Hydroiden der arktischen Meere. Fauna Arctica 5: 127-248.
4. Broch H (1914) Stylasteridae. The Danish Ingolf-Expedition 5(5): 1-26.
5. Cairns SD, Lindner A (2011) A revision of Stylasteriidae (Cnidaria, Hydrozoa, Filifera) from Alaska and adjacent waters. ZooKeys 158: 1-88.
6. Calder DR (1970) Thecate hydroids from the shelf waters of Northern Canada. Journal of the Fisheries Research Board of Canada 27(9): 1501-1547.
7. Calder DR (1972) Some athecate hydroids from the shelf waters of Northern Canada. Journal of the Fisheries Research Board of Canada 29(3): 217-228.
8. Clark SF (1877) Report on the hydroids collected on the coast of Alaska and the Aleutian Islands, by W. H. Dall, U.S. coast survey, and party, from 1871 to 1874 inclusive. Proceedings of the Academy of Natural Sciences of Philadelphia 28: 209-238.
9. Cooney RT (1981) Bering Sea zooplankton and micronekton communities with emphasis on annual production. In: Hood DW, Calder JA, eds. The eastern Bering Sea shelf: Oceanography and Resources. Vol. 2. Seattle: University of Washington Press. pp. 947-974.
10. Dunbar MJ (1942) Marine macroplankton from the Canadian Eastern Arctic. II. Medusae, Siphonophora, Ctenophora, Pteropoda and Chaetognatha. Can J Res 20(D): 71-77.
11. Fisher WK (1938) Hydrocorals of the North Pacific Ocean. In: [Proceedings of the United States National Museum](http://www.google.pl/search?hl=pl&tbo=p&tbm=bks&q=bibliogroup:"Proceedings+of+the+United+States+National+Museum"&source=gbs_metadata_r&cad=4), Tom 84. U.S. Government Printing Office. pp. 493-554.
12. Fraser CM (1944) Hydroids of the Atlantic coast of North America. The University of Toronto Press, Toronto. 451 p.
13. Grainger EH (1965) Zooplankton from the Arctic Ocean and adjacent Canadian waters. Journal of the Fisheries Research Board of Canada 22(2): 543-564.
14. Grainger EH, Grohe K (1975) Zooplankton data from the Beaufort Sea, 1951 to 1975. Fish Mar Serv Res Dev Tech Rep 591. 54 pp.
15. Hand C, Kan LB (1961) The medusae of Chukchi and Beaufort seas of the arctic ocean including the description of a new species of *Eucodonium* (Hydrozoa: Anthomedusae). Arctic Institute of North America; technical papers 6: 1-23.
16. Jäderholm E (1916) Die Hydroiden des Eisfjords. Bihang till Kungliga Svenska Vetenskapsakademiens Hand-lingar 54(4): 1-14.
17. Kosobokova K, Hirche H-J (2000) Zooplankton distribution cross the Lomonosov Ridge, Arctic Ocean: species inventory, biomass and vertical structure. Deep Sea Res Part 1 Oceanogr Res Pap 47: 2029-2060.
18. Kosobokova K, Hanssen H, Hirche H-J, Knickmeier K (1998) Composition and distribution of zooplankton in the Laptev Sea and adjacent Nansen Basin during summer, 1993. Polar Biol 19: 63-76.
19. Kosobokova K, Hopcroft RR, Hirche H-J (2011) Patterns of zooplankton diversity through the depths of the Arctic’s central basin. Mar Biodivers 41: 29-50.
20. Kramp P (1932) Hydroids collected in West-Greenland Fjords in 1911 and 1912. Meddelelser om Grønland 91: 1-35.
21. Kramp P (1942) Siphonophora. The Godthaab Expedition 1928. Meddelelser om Grønland 80(8): 3-24.
22. Kramp P (1943) The zoology of East Greenland. Hydroida. Meddelelser om Grønland 121: 1-52.
23. Kramp P (1947) Medusae. Part III. Trachylina and Scyphozoa, with zoogeographical remarks on all the medusae of the northern Atlantic. Danish Ingolf Expedition 5: 1-66.
24. Kramp P (1959) The Hydromedusae of the Atlantic Ocean and adjacent waters. Dana-Report 46: 1-283.
25. Kramp P (1961) Synopsis of the medusae of the world. J Mar Biol Assoc U.K. 40: 1-469.
26. Mapstone GM (2009) Siphonophora (Cnidaria: Hydrozoa) of Canadian Pacific waters. NRC Research Press, Ottawa, Ontario, Canada. 302 p.
27. Margulis RYa (1978) The distribution of siphonophores in the western North Atlantic in summer of 1974. Vestnik Moskovskogo Universiteta, Ser. XVI Biologiya 3: 1-11.
28. Margulis RYa (1982) A new *siphonophore Rudjakovia plicata* gen. n., sp. n., (Coelenterate, Hydrozoa) from the Polar Basin and some notes on other siphonophores. Zoological Journal 61: 440-444.
29. Marktanner-Turneretscher G (1895) Hydroiden von Ostspitsbergen. Zoologische Jahrbücher, Abteilung Systematik und Geographie der Tiere 8: 391-438.
30. Miglietta MP (2006) *Hydractinia antonii* sp. nov.: a new, partially calcified hydractiniid (Cnidaria: Hydrozoa: Hydractiniidae) from Alaska. J Mar Biol Assoc U.K. 86: 993-996.
31. Motoda S, Minoda T (1974) Plankton of the Bering Sea. In: Hood DW, Kelly J, eds. Oceanography of the Bering Sea; Proceedings of the International Symposium for Bering Sea Studies, Hakodate, Japan, 21 January to 4 February, 1972. University of Alaska, Fairbansk. pp. 207-241.
32. Naumov DV (1969) Hydroids and Hydromedusae of the USRR. Jerusalem: Israel Program for scientific translation. 660 p.
33. Nutting CC (1901) Papers from the Harriman Alaska Expedition. XXI. The hydroids. Proceedings of the Washington Academy of Science 3: 157-216.
34. Petersen KW (1990) Evolution and taxonomy in capitate hydroids and medusae (Cnidaria: Hydrozoa). Zool J Linn Soc 100: 101-231.
35. Piraino S, Bluhn BA, Gradinger R & Boero F (2008) Sympagohydra tuuli gen. nov. and sp. nov. (Cnidaria: Hydrozoa) a cool hydroid from the Arctic sea ice. J Mar Biol Assoc U.K. 88: 1637-1641.
36. Pugh PR, Pagès F (1995) Is *Lensia reticulata* a diphyine species (Siphonophorae, Calycophora, Diphyidae)? A re-description. Scientia Marina 59(2): 181-192.
37. Raskoff KA, Purcell JE, Hopcroft RR (2005) Gelatinous zooplankton of the Arctic Ocean: in situ observations under the ice. Polar Biol 28: 207-217.
38. Raskoff KA (2010) *Bathykorus bouilloni*: a new genus and species of deep-sea jellyfish from the Arctic Ocean (Hydrozoa, Narcomedusae, Aeginidae). Zootaxa 2361: 57-67.
39. Raskoff KA, Hopcroft RR, Kosobokova KN, Purcell JE, Youngbluth M (2010) Jellies under ice: ROV observations from the 2005 Hidden Ocean Expedition. Deep Sea Res 2 Top Stud Oceanogr 57: 111-126 .
40. Ronowicz M (2007) Benthic hydroids (Cnidaria: Hydrozoa) from Svalbard waters - biodiversity and distribution. J Mar Biol Assoc U.K. 87: 1089-1094.
41. Ronowicz M, Schuchert P (2007) *Halecium arcticum*, a new hydroid from Spitsbergen (Cnidaria: Hydrozoa). Zootaxa 1549: 55-62.
42. Ronowicz M, **Włodarska-Kowalczuk M, Kukliński P** (2013a) Hydroid epifaunal communities in Arctic coastal waters (Svalbard): effects of substrate characteristics. Polar Biol 36: 705-718.
43. Ronowicz M, Włodarska-Kowalczuk M, Kukliński P (2013b) Depth- and substrate-related patterns of species richness and distribution of hydroids (Cnidaria, Hydrozoa) in Arctic coastal waters ( Svalbard ). Mar Ecol 34: 165-176.
44. Schuchert P (2001) Hydroids of Greenland and Iceland (Cnidaria, Hydrozoa). Meddelelser om Grønland, Bioscience 53: 1-184.
45. Schuchert P (2006) The European athecate hydroids and their medusae (Hydrozoa, Cnidaria): Capitata Part 1. Rev Suisse Zool 113: 325-410.
46. Schuchert P (2008a) The European athecate hydroids and their medusae (Hydrozoa, Cnidaria): Filifera Part 3. Rev Suisse Zool 115: 221-302.
47. Schuchert P (2008b) The European athecate hydroids and their medusae (Hydrozoa, Cnidaria): Filifera Part 4. Rev Suisse Zool 115: 677-757.
48. Schuchert P (2010) The European athecate hydroids and their medusae (Hydrozoa, Cnidaria): Capitata Part 2. Rev Suisse Zool 117: 337-555.
49. Schuchert P (2012) North-West European Athecate Hydroids and their Medusae. Synopses of the British Fauna (New Series) 59. 164 p.
50. Shih CT, Figueira AJG, Grainger EH (1971) A synopsis of Canadian marine zooplankton. Bulletin of the Fisheries Research Board of Canada 176: 1-264.
51. Shirley WD, Leung Y-M (1970) Medusae of the Central Arctic. In: Kobayashi HE, ed. Taxonomic Guides to Arctic Zooplankton II [Technical report no 3, 1960-1970]. University of Southern California Los Angeles, Department of Biological Sciences, Los Angeles.
52. Stepanjants SD (1967) Siphonophores of the seas of the USSR and the northern part of the Pacific Ocean. Opredeliteli po Faune SSSR 96. 216 p.
53. Stepanjants SD (1989) Hydrozoa of the Eurasian Arctic Seas. In: Herman Y, ed. The Arctic Seas Climatology, Oceanography, and Biology. New York: Van Nostrand Reinhold. pp. 397-430.
54. Stepanjants SD (1994) Hydrozoa of the East Siberian Sea. Issledovaniya Fauny Morei 48: 116-142.
55. Stepanjants SD (2001) Subphylum Medusozoa. Classes Hydrozoa, Siphonophora, Scyphozoa. In: Sirenko BI, ed. List of species of free-living Invertebrates of Eurasian Arctic Seas and Adjacent deep waters. Issledovania Fauni Morey 51(59): 31- 36.
56. Stepanjants SD, Kosobokova KN (2006) Medusae of the genus *Rhabdoon* Hydrozoa: Anthomedusae: Tubularioidea in the Arctic Ocean. Mar Biol Res 2(6): 388-397.
57. Stepanjants SD, Svoboda A (2008). The genus *Gymnogonos* (Anthoathecata: Capitata: Corymorphidae) - redescription of known species and description of a new species from the North Pacific. J Mar Biol Assoc U.K. 88: 1619-1629.
58. Thiel ME (1932) Übersicht über die Hydromedusen der Arktis. Fauna Arctica 6: 119-158.
59. Uchida T (1969) Medusae from the Arctic Ocean. Publications of the Seto Marine Biological Laboratory 17(4): 285-287.
60. van Soest RWM (1973) Planktonic coelenterates collected in the North Atlantic Ocean. Bijdragen tot de Dierkunde 43(1): 119–125.
61. Voronkov A, Stepanjants SD, Hop H (2010) Hydrozoan diversity on hard bottom in Kongsfjorden, Svalbard. J Mar Biol Assoc U.K. 90: 1337-1352.
62. Węsławski JM, Kwaśniewski S, Wiktor J (1991) Winter in a Svalbard fiord ecosystem. Arctic 44(2): 115-123.
63. Yashnov VA (1948) Hydromedusae. In: Gaevskoy NL, ed. Check List Fauna Flora northern seas U.S.S.R., Moscow. pp. 65-74.
64. Zelickman EA (1972) Distribution and ecology of the pelagic hydromedusae, siphonophores and ctenophores of the Barents Sea, based on perennial plankton collections. Mar Biol 17: 256–264.
